# Electrochemical Detection of Cd^2+^, Pb^2+^, Cu^2+^ and Hg^2+^ with Sensors Based on Carbonaceous Nanomaterials and Fe_3_O_4_ Nanoparticles

**DOI:** 10.3390/nano14080702

**Published:** 2024-04-18

**Authors:** Ancuța Dinu (Iacob), Alexandra Virginia Bounegru, Catalina Iticescu, Lucian P. Georgescu, Constantin Apetrei

**Affiliations:** Department of Chemistry, Physics and Environment, Faculty of Sciences and Environment, “Dunărea de Jos” University of Galati, 47 Domneasca Street, 800008 Galați, Romaniacatalina.iticescu@ugal.ro (C.I.); lucian.georgescu@ugal.ro (L.P.G.)

**Keywords:** heavy metals, sensor, square-wave stripping voltammetry, water

## Abstract

Two electrochemical sensors were developed in this study, with their preparations using two nanomaterials with remarkable properties, namely, carbon nanofibers (CNF) modified with Fe_3_O_4_ nanoparticles and multilayer carbon nanotubes (MWCNT) modified with Fe_3_O_4_ nanoparticles. The modified screen-printed electrodes (SPE) were thus named SPE/Fe_3_O_4_-CNF and SPE/Fe_3_O_4_-MWCNT and were used for the simultaneous detection of heavy metals (Cd^2+^, Pb^2+^, Cu^2+^ and Hg^2+^). The sensors have been spectrometrically and electrochemically characterized. The limits of detection of the SPE/Fe_3_O_4_-CNF sensor were 0.0615 μM, 0.0154 μM, 0.0320 μM and 0.0148 μM for Cd^2+^, Pb^2+^, Cu^2+^ and Hg^2+^, respectively, and 0.2719 μM, 0.3187 μM, 1.0436 μM and 0.9076 μM in the case of the SPE/ Fe_3_O_4_-MWCNT sensor (following optimization of the working parameters). Due to the modifying material, the results showed superior performance for the SPE/Fe_3_O_4_-CNF sensor, with extended linearity ranges and detection limits in the nanomolar range, compared to those of the SPE/Fe_3_O_4_-MWCNT sensor. For the quantification of heavy metal ions Cd^2+^, Pb^2+^, Cu^2+^ and Hg^2+^ with the SPE/Fe_3_O_4_-CNF sensor from real samples, the standard addition method was used because the values obtained for the recovery tests were good. The analysis of surface water samples from the Danube River has shown that the obtained values are significantly lower than the maximum limits allowed according to the quality standards specified by the United States Environmental Protection Agency (USEPA) and those of the World Health Organization (WHO). This research provides a complementary method based on electrochemical sensors for in situ monitoring of surface water quality, representing a useful tool in environmental studies.

## 1. Introduction

In recent years, environmental heavy metal (HM) pollution has increased considerably due to major industrialization in several countries of the world [1,2,3,4]. High concentrations of heavy metals can sometimes be found in water, soil, or air, a situation that can lead to serious health problems for vegetation and wildlife, as well as humans [5,6,7,8].

Heavy metals are those metallic elements with a density greater than 4.5 g·cm^−3^, such as Cd, Cr, Hg, Pb, Cu or As, all of which are known to have high toxicity, long half-life and non-biodegradability [9,10,11]. These pollutants come mainly from mining and industrial activities, wastes from mining, smelting, metal refining and coal burning operations [12,13,14]. When heavy metals enter the environment, they come into direct contact with living organisms and begin to accumulate causing extremely dangerous manifestations such as damage to the digestive, renal, neuronal, cardiovascular, reproductive, visual analyzer systems and even molecular changes in the DNA [15,16,17,18]. It is worth mentioning that there are also HMs that are essential for the body and maintenance of health, such as iron, zinc and copper, but even these can become toxic at high concentrations [19,20]. Therefore, continuous, rapid, sensitive and accurate monitoring of HMs in environmental and food samples is necessary [21,22,23].

Conventional methods for tracing metal detection at low concentrations include atomic absorption spectroscopy (AAS) [24,25,26,27], neutron activation analysis and inductively coupled plasma–optical emission spectrometry (ICP–OES) [28,29,30,31], capillary electrophoresis (CE) [32], ion chromatography ultraviolet–visible spectroscopy (IC–UV–Vis) [33,34], inductively coupled plasma mass spectrometry (ICP–MS) [35,36,37,38,39], microwave field hydrothermal method [40,41], X-ray fluorescence spectroscopy (XFS) [42,43,44], etc. Although these methods have the advantage of high sensitivity and selectivity, there are also some aspects that may limit their use in routine analysis and real-time monitoring of HM ion concentration, such as the high cost of instruments, laborious sample preparation steps, the need for a professional experienced in the application of this technique and laborious preconcentration procedures. Moreover, the equipment is extremely expensive and bulky, which is why it cannot be used for in situ detection [18].

On the other hand, recent research has shown that electrochemical techniques used for heavy metal detection have made significant progress. Recent approaches use electrodes modified with silicon-based materials [45], polymers [46,47], metal nanoparticles [48,49] or carbon-based materials (graphene, carbon nanotubes or carbon nanofibers) [50,51,52] to enhance the sensitivity and selectivity of HM detection systems. The large surface area, the possibility of functionalization and the extraordinary quantum-mechanical properties of nanomaterials make them suitable and promising for electrode modification. The improved performance of nanomaterial-based sensors comes from the nanoscale electrode surface modification that provides increased catalytic activity, improved conductivity, larger active surface area and very good electrode sensitivity [53,54,55]. Carbon nanotubes and carbon nanofibers are often used to modify electrodes used in heavy metal detection with excellent results [56,57,58,59]. Although both exhibit very good electrocatalytic properties, there are still some differences in terms of shape, size, sensitivity, selectivity and stability that can be amplified or diminished by their functionalization with different nanocomposites, e.g., oxides of transition metals. Transition metal oxides provide a large surface area for the adsorption of analyte molecules to the electrode surface and speed up the charge transfer process. They have higher specific capacitance, high electrical conductivity, higher energy density than carbon materials and better chemical stability. At the same time, carbon-based materials have a large surface with numerous active sites. Also, the carbon matrix acts as a supporting medium for the homogeneous dispersion of nanoparticles, prevents their aggregation and gives a large surface area to volume ratio, enhancing the electrocatalytic process. Carbon matrices are well-known for their large surface area, tensile mechanical strength, rapid rate of electron transfer, exceptionally high electrical and thermal conductivities, which improve the electrochemical process [60,61].

Ferroferric oxide, Fe_3_O_4_ (iron (II, III) oxide), is a material made of magnetic particles with unique properties such as magnetism and chemical reactivity. Fe_3_O_4_ has been widely used in the chemical industry due to its chemical stability, easy preparation, excellent catalytic performance and low toxicity [62,63,64]. These properties make Fe_3_O_4_ a very useful nanocomposite in various applications, including the functionalization of carbon materials for the detection of heavy metals such as lead, cadmium, mercury and copper. However, due to the high surface energy, Fe_3_O_4_ nanoparticles show a tendency to form aggregates, leading to a significant decrease in active sites [65]. This drawback could be avoided by using a conductive material as a support such as carbon nanotubes or carbon nanofibers, due to their remarkable properties [66]. The introduction of a carbon nanomaterial alongside ferroferric oxide nanoparticles can reduce the formation of Fe_3_O_4_ aggregates, increasing the active surface area available for detection, electron transfer rate and catalytic activity through synergy, and, thus, can improve the detection sensitivity [63].

By using a sensor functionalized with Fe_3_O_4_, efficient detection of heavy metal presence can be achieved through the method of anodic stripping voltammetry. This process involves modifying the sensor’s surface with Fe_3_O_4_ nanoparticles, creating a surface with an affinity for heavy metals. The heavy metals in the solution are then attracted and deposited onto the sensor’s surface by applying an appropriate voltage. In the final stage, through the application of a voltammetric method (such as CV, DPV, SWV), the oxidation of the heavy metals on the sensor’s surface takes place, leading to an increase in the electric current intensity. This increase is proportional to the initial concentration of heavy metals and provides a sensitive and precise means of detection.

The aim of this study is to investigate the effectiveness and accuracy of electrochemical sensors modified with Fe_3_O_4_ nanoparticles and carbon nanofibers for the simultaneous detection and quantification of heavy metal ions in standard solutions of Cd^2+^, Pb^2+^, Cu^2+^ and Hg^2+^ using square-wave anodic stripping voltammetry. This research adds value to the development of rapid, precise and sensitive methods for detecting heavy metal pollution, with the potential to contribute to environmental monitoring and public health protection.

The novelty of this study resides in the optimized, rapid and easy method of synthesizing Fe_3_O_4_ nanoparticles, utilizing only a few reagents, as well as in the development of new nanocomposites based on carbon nanomaterials. These innovations were utilized to modify two screen-printed electrodes. In this study, the characterization and comparison of two electrochemical sensors modified with Fe_3_O_4_ and carbon nanofibers (SPE/Fe_3_O_4_-CNF) and multilayer carbon nanotubes (SPE/Fe_3_O_4_-MWCNT) were performed on the qualitative and quantitative, simultaneous electrochemical detection of heavy metal ions in standard solutions of the metal ions Cd^2+^, Pb^2+^, Cu^2+^ and Hg^2+^ using square-wave anodic stripping voltammetry. The results are compared with those obtained using the standard addition method to confirm the feasibility of the voltammetric method.

## 2. Materials and Methods

### 2.1. Reagents and Solutions

The supporting electrolyte used for analytical measurements was an acetate buffer solution prepared from sodium acetate and acetic acid purchased from Sigma-Aldrich, St. Louis, MO, USA. The amounts of reagents varied according to the desired pH (4, 4.5, 5, 5.5) and the exact value was determined using the WTW pH meter (Weilheim, Germany). Potassium chloride and potassium ferro- and ferricyanide (Sigma-Aldrich, St. Louis, MO, USA) were used to prepare the solution in which EIS analysis was performed for the characterization of the modified sensors. Multilayer carbon nanotube (MWCNT) powder, carbon nanofibers (CNFs) (Sigma-Aldrich, St. Louis, MO, USA), FeCl_3_·6H_2_O (Carlo Erba Reagents GmbH, Cornaredo, Milan, Italy), ascorbic acid (Sigma-Aldrich, St Louis, MO, USA), hydrazine (LOBA Feinchemie GmbH, Fehrgasse, Austria), ethanol (International Laboratory SRL, Cluj Napoca, Romania), dimethylformamide (DMF) (Sigma-Aldrich, St. Louis, MO, USA) and ultrapure water (obtained with a Milli-Q system—Millipore, Bedford, MA, USA) were used for this study.

The heavy metal salts, Cu(NO_3_)_2_, (CH_3_COO)_2_Pb, Hg(NO_3_)_2_ and (CH_3_COO)_2_Cd·2H_2_O (Reactiv Plus SRL, Bucharest, Romania), were weighed and solubilized in acetate buffer solution to a concentration of 10^−4^ M. Bismuth nitrate (Reactiv Plus SRL, Bucharest, Romania) was then added to the solution for signal optimization at a concentration of 0.3 mg/L.

The actual water samples were taken from the Danube River on the same day from different collection points. Before analysis, the samples were filtered through filter paper to remove possible impurities or visible residues.

### 2.2. Equipment

Electrochemical measurements were carried out using a potentiostat/galvanostat Autolab PGSTAT 302N controlled by Nova 2.1 software, to which an electrochemical cell with a volume of 50 mL was connected. The reference electrode was Ag/AgCl (Princeton, Applied Research), and the auxiliary electrode was platinum wire. The 4 mm diameter screen-printed working electrode was connected to the potentiostat/galvanostat, represented by the modified screen-printed sensors developed in this study. An ultrasonic bath was used for the dissolution of compounds, homogenization of solutions and dispersion of nanoparticles and nanofibers. To characterize the modified sensors and validate the results, FT-IR measurements in attenuated total reflectance (ATR) mode were performed. The ATR crystal made of ZnSe was cleaned with ultrapure water and isopropanol after each measurement. Buffer solutions were adjusted to the desired pH using a glass pH electrode connected to a WTW pH meter (Weilheim, Germany). Cencom II centrifuge was used in the separation process of Fe_3_O_4_-MWCNT and Fe_3_O_4_-CNF nanocomposites.

### 2.3. Synthesis of Fe_3_O_4_-MWCNT and Fe_3_O_4_-CNF Nanocomposites

In the first step, Fe_3_O_4_ nanoparticles were synthesized using a mixture consisting of 0.3 g FeCl_3_·6H_2_O, 0.5 g ascorbic acid, 10 mL hydrazine and 50 mL ultrapure distilled water. The mixture was dried in the oven for 8 h at 180 °C. During the 8 h, the mixture was rinsed twice with ethanol and water. In the second step, 25 mg Fe_3_O_4_ and 100 mg MWCNT or CNF were dispersed in 10 mL ethanol and centrifuged for 5 min, then dried at 150 °C for 2 h.

While the synthesis process was carefully controlled to ensure the formation of Fe_3_O_4_ nanoparticles, the focus of this research was on the development and application of nanocomposites and the synthesis method for Fe_3_O_4_ nanoparticles was based on established protocols [67,68].

### 2.4. Construction of Electrochemical Sensors

**Figure 1:**
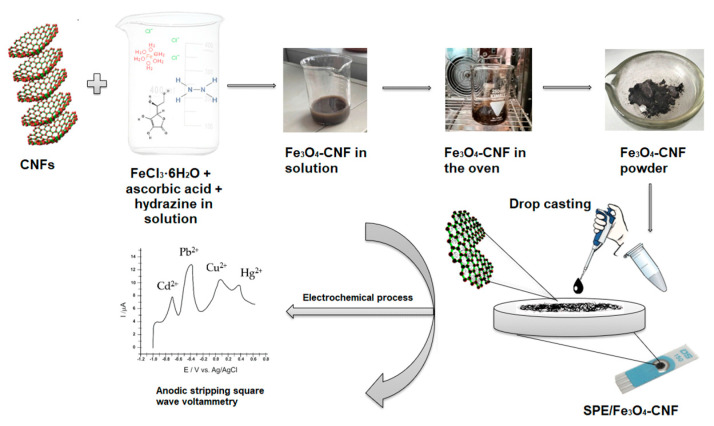
Scheme of the SPE/Fe_3_O_4_-CNF fabrication.

The suspension with catalytic properties was prepared by dispersing 10 mg Fe_3_O_4_-MWCNT, Fe_3_O_4_-CNF mixture in 500 μL water and dimethylformamide in a 1:1 ratio. The mixture was sonicated for 30 min for good homogenization. A volume of 10 μL was deposited on the SPE surface by the drop-casting technique in two steps with a drying break of 1 h. The added suspension volume was optimized. For the preliminary analyses, MWCNT-only and CNF-only screen-printed sensors were also constructed using the same technique and suspension volume. All modified sensors were kept at room temperature. The steps in the SPE/Fe_3_O_4_-CNF sensor construction are schematically represented in Figure 1.

### 2.5. Methods of Analysis

Electrochemical methods (electrochemical impedance spectroscopy) and spectrometric methods (Fourier transform infrared spectroscopy) were used to characterize the modified sensors. The electrochemical behavior of the modified sensors in an aqueous solution with K_4_[Fe(CN)_6_] 10^−3^ M and KCl 10^−1^ M was evaluated by cyclic voltammetry (CV). Square-wave anodic stripping voltammetry (SWASV) was employed for the detection of heavy metal ions, their calibration curve and quantification in real samples.

### 2.6. Statistical Data Processing Methodology

Various statistical data processing techniques were employed for the analysis and interpretation of data obtained from the electrochemical study and for evaluating the performance of the developed sensors. These techniques include linear regression, calculation of standard deviation, recovery calculation, coefficient of variation and interpretation of the obtained electrochemical parameters. Excel software (version 2401) and the Data Analysis Toolbox were utilized for the statistical analysis of these data.

## 3. Results and Discussion

### 3.1. Characterization of the Modified Surface

Electrochemical impedance spectroscopy (EIS) was used to characterize the modified active surface of the new electrochemical sensors. This technique is based on measuring the electrical impedance of an electrochemical cell as a function of frequency when an alternating voltage is applied. EIS can provide information about the electrochemical properties of the modified surface, such as resistance, capacitance and the kinetics of electron transfer, but it does not directly provide information about the sensitivity and selectivity of an electrode, which are analytical parameters.

**Figure 2:**
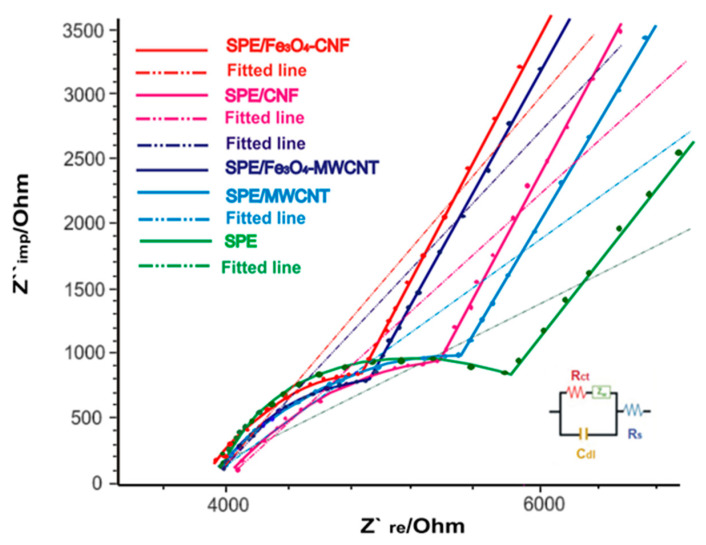
Nyquist plots of EIS and fitted line for SPE/Fe_3_O_4_-CNF (red line), SPE/CNF (pink line), SPE/Fe_3_O_4_-MWCNT (dark blue line), SPE/MWCNT (blue line) and SPE (green line) in 10^−1^ M KCl and 10^−3^ M [Fe_4_(CN)_6_]^3−/4−^ for a frequency range of 0.1 Hz to 10^6^ Hz, amplitude 5 mV. Inset: Equivalent circuit is applied to fit the impedance spectra.

In this case, the electrochemical processes taking place at the interface of the modified working electrodes and the electrolyte chosen for analysis (in this case, 10^−3^ M K_3_[Fe(CN)_6_]/K_4_[Fe(CN)_6_] and 10^−1^ M KCl in a 1:1 ratio) were fitted using an equivalent circuit involving resistors, capacitors and inductors. The Randles equivalent circuit is a commonly used model in electrochemical impedance spectroscopy to represent the electrochemical processes occurring at the electrode interface. It shows, in a simplified way, the solution resistance (R_s_), the capacitance of the double layer at the electrode surface (C_dI_), the charge transfer resistance (R_ct_) and the Warburg resistance (Z_w_) [69]. According to Figure 2, the Nyquist diagrams obtained in this study (in the form of a semicircle) correspond to a simulated Randles circuit.

In the case of modified sensors, the electron transfer resistance (R_ct_) is being evaluated, with a higher value indicating lower conductivity. The presence of the Fe_3_O_4_ nanoparticles leads to lower electron transfer resistance and, thus, better electrocatalytic properties. As expected, the R_ct_ value was 5353.7 Ω for the SPE/CNF electrode, and, for the SPE/Fe_3_O_4_-CNF, it decreases to a value of 4849.2 Ω. The same behavior is observed for SPE/MWCNT (5505.67 Ω) and SPE/Fe_3_O_4_-MWCNT (4858.16 Ω). R_ct_ values of the modified sensors are lower than that of the unmodified screen-printed electrode (SPE) (R_ct_ = 5805 Ω). It is worth mentioning that SPE/Fe_3_O_4_-CNF has the lowest value of electron transfer resistance, so it can be assumed that the structure and size of carbon nanofibers, together with Fe_3_O_4_ nanoparticles, enhance their electrical properties, favoring a more sensitive detection.

**Figure 3:**
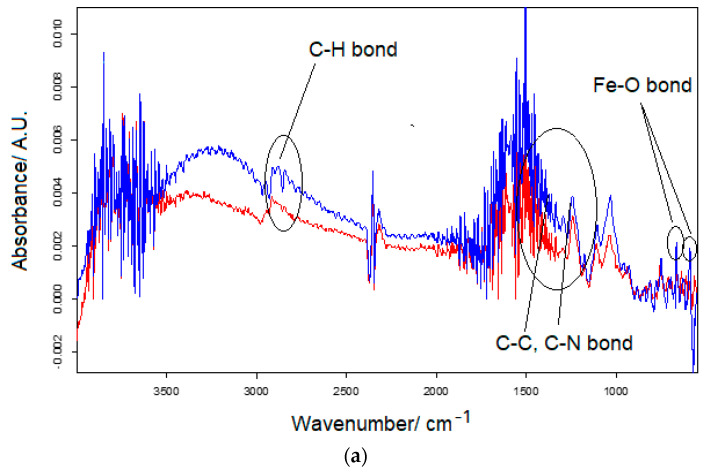

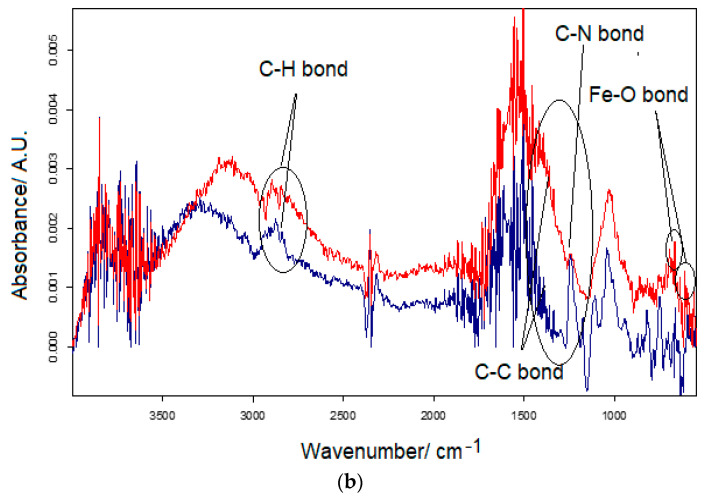
FT-IR spectra for (**a**) SPE/CNF (red line), SPE/Fe_3_O_4_-CNF (blue line), and (**b**) SPE/Fe_3_O_4_-MWCNT (blue line).

FT-IR spectroscopy was used to evaluate the main functional groups present in the four composite nanomaterials deposited on the screen-printed electrodes (Figure 3). The characteristic vibrational stretching associated with Fe-O bonds, where Fe_3_O_4_ nanoparticles exist, is observed at wavelengths 585 and 640 cm^−1^ in the case of SPE/Fe_3_O_4_-MWCNT, respectively, at 560 and 625 cm^−1^ in the case and SPE/Fe_3_O_4_-CNF. These bands correspond to Fe-O (Fe (II) and/or Fe (III)) in octahedral and tetrahedral geometries, as mentioned in the literature [70]. In all four spectra, bending vibrations in the range 1500–1700 cm^−1^ and stretching vibrations in the range 3250–3300 cm^−1^ of the O-H groups occur. Finally, the existence of carbonaceous nanomaterials is inferred by the C-H stretching bands between 2850–2925 cm^−1^ and the vibrations of the C-C and C-N groups in the range 1200–1500 cm^−1^.

### 3.2. Electrochemical Behavior of SPE/Fe_3_O_4_-MWCNT and SPE/Fe_3_O_4_-CNF in Potassium Ferrocyanide Solution

An intriguing observation in our study pertains to the electrochemical behavior of the MWCNTs composite compared to CNFs. Despite the higher intrinsic conductivity of MWCNTs, the composite exhibited lower electron transfer kinetics and surface area. Several factors contribute to this behavior. Firstly, the nature of the interaction between MWCNTs and Fe_3_O_4_ nanoparticles may differ from that with CNFs, affecting the overall conductivity of the composite. Secondly, the dispersion quality of MWCNTs in the composite may be different from that of CNFs. Any aggregation can reduce the effective surface area and hinder electron transfer. Thirdly, structural variations between MWCNTs and CNFs, such as the number of layers, alignment and defects, can lead to differences in electron transfer kinetics and surface area. Lastly, the performance of the composite is influenced by the synergistic effects of its components. The combination of Fe_3_O_4_ with CNFs may result in a more favorable synergy for electron transfer compared to the combination with MWCNTs. This detailed analysis provides insights into the complex behavior of the CNFs composite and enhances our understanding of its electrochemical properties in comparison to MWCNTs.

**Figure 4:**
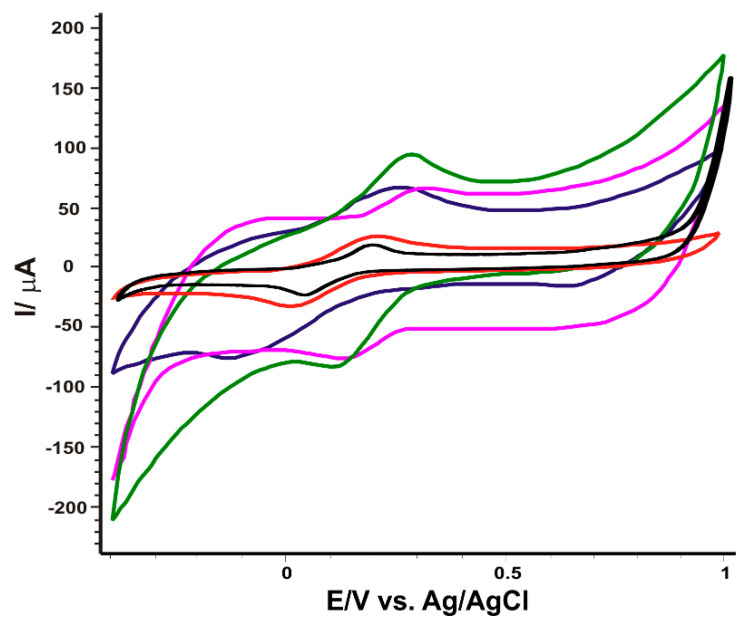
The cyclic voltammograms of SPE (black line), SPE/CNF (red line), SPE/Fe_3_O_4_-CNF (green line), SPE/MWCNT (violet line) and SPE/Fe_3_O_4_-MWCNT (blue line) immersed in K_4_[Fe(CN)_6_] 10^−3^ M-KCl 10^−1^ M solution. Scan rate 0.1 V·s^−1^.

The electrochemical behavior of SPE bare and the four modified sensors (SPE/MWCNT, SPE/CNF, SPE/Fe_3_O_4_-MWCNT, SPE/Fe_3_O_4_-CNF) was studied in a solution of K_4_[Fe(CN)_6_] 10^−3^ M-KCl 10^−1^ M. The potential range was between −0.4 and +1.0 V, and the scan rate was 0.1 V·s^−1^. Figure 4 shows the redox process of potassium ferrocyanide, well-highlighted with each electrode. Oxidation and reduction peaks are present in each situation, proving a quasi-reversible redox process. However, the intensity and potential of the peaks are slightly different depending on the sensor used, which shows the differences imprinted by the modifications of the active surface.

As can be seen in Table 1, all modified sensors show a value of the ratio I_pc_/I_pa_ very close to 1, indicating optimal peak reversibility. Although the oxidation peak intensities of SPE/MWCNT and SPE/Fe_3_O_4_-MWCNT sensors have very close values, the latter exhibits a lower oxidation potential, suggesting better selectivity, likely due to the presence of Fe_3_O_4_ nanoparticles on the active surface of SPE/Fe_3_O_4_-MWCNT. On the other hand, SPE/Fe_3_O_4_-CNF shows the highest peaks, an I_pc_/I_pa_ ratio close to 1 and an optimal E_1/2_ value (fairly close to the theoretical E_1/2_ value (0.36 V) obtained from the hydrogen electrode in the [Fe(CN)_6_]^4−^-[Fe(CN)_6_]^3−^ redox process [71]), suggesting favorable dynamics of the redox process. The slightly lower value of ΔE obtained by the SPE/Fe_3_O_4_-CNF sensor suggests faster kinetics and improved reversibility of the redox process at the electrode interface. While the difference in performance between the modified SPE/CNF and SPE/Fe_3_O_4_-CNF sensors, and between SPE/MWCNT and SPE/Fe_3_O_4_-MWCNT, may not be substantial, it is noteworthy. The attachment of colloidal particles on a given surface and their interaction is a complex process, dependent on several parameters such as surface chemical processes, colloidal particle size, presence of ligands and type of dispersant [72]. Therefore, the observed variations in electrochemical response can be attributed to differences in the interaction between Fe_3_O_4_ and CNFs, influenced by the size and shape of CNFs and the resulting synergistic effects between nanocomposites. To calculate the active surface area of the modified sensors, the kinetics of the redox process of 10^−3^ M potassium ferrocyanide–10^−1^ M KCl was studied by applying several scanning rates (0.1–0.5 V·s^−1^). Following the measurements, a linear dependence between the intensity of the oxidation peak and the square of the scan rate is found in each case. This result shows that the electrochemical process is controlled by the diffusion of the ferrocyanide ion at the surface of the working electrode. Therefore, knowing the diffusion coefficient of K_4_[Fe(CN)_6_] (7.26 × 10^−6^ cm^2^·s^−1^ [73], from the Randles–Sevcik equation [74], the active surface areas of the four sensors were found, and the values are shown in Table 2.

It can be seen in Table 2 that the active surface area increases significantly after the addition of Fe_3_O_4_ in the modifying nanocomposite material, especially in the case of SPE/Fe_3_O_4_-CNF, where the roughness factor is 2.12.

Nevertheless, through the analysis of the log(I) vs. log(v) graphs for the modified electrodes, a broader perspective emerges regarding the influence of chemical interactions in the redox process of potassium ferrocyanide. The slope values of 0.73 for SPE/Fe_3_O_4_-CNF and 0.75 for SPE/Fe_3_O_4_-MWCNT indicate significant deviations from the theoretical model (0.5) [75], suggesting a pronounced influence of chemical species in the redox reaction for both modified electrodes. This underscores their enhanced efficiency in capturing and recognizing redox species, thereby facilitating further applications in sensitive and selective analytical detection. The subtle differences in slope values between the two types of electrodes can be attributed to specific surface variations.

Since the addition of Fe_3_O_4_ nanoparticles helped to improve the responses in the preliminary studies and the nanocomposite showed up on the active surface of the electrodes by both FT-IR and EIS methods, only the modified SPE/Fe_3_O_4_-CNF and SPE/Fe_3_O_4_-MWCNT sensors were used for heavy metal ion detection studies.

### 3.3. Electrochemical Detection of Heavy Metals Using SPE/Fe_3_O_4_-MWCNT and SPE/Fe_3_O_4_-CNF

Square-wave anodic stripping voltammetry (SWASV) involves applying a variable voltage to the working electrode that varies rapidly between two values. This potential variation leads to a variation in electric current, which can be measured and recorded. This is because heavy metals deposited on the surface of the electrode have a tendency to oxidize at the anode, leading to an increase in the electric current. The magnitude of the electric current depends on the concentration of heavy metals in the solution [76,77].

A stock solution of Cd^2+^, Hg^2+^, Cu^2+^ and Pb^2+^ in equal concentrations of 10^−4^ M in 10^−1^ M acetate buffer solution was prepared for heavy metal detection analysis.

After immersion of the electrode in the stock solution, parameters were set for the electrodeposition step, for square-wave voltammetry measurements, and for the desorption step of heavy metals from the surface of the working electrodes.

**Figure 5:**
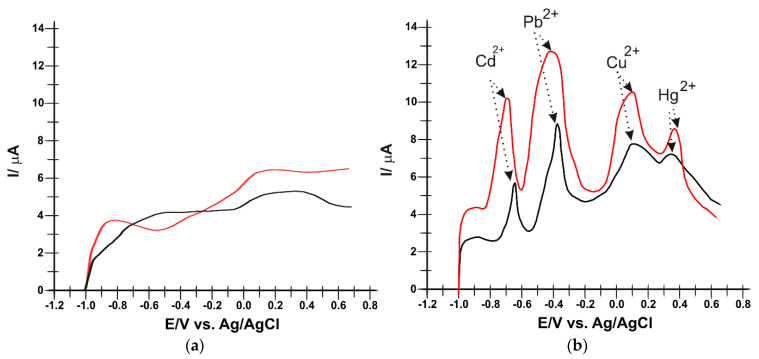
SWASV of SPE/Fe_3_O_4_-CNF (red line) and SPE/Fe_3_O_4_-MWCNT (black line) in (**a**) 10^−1^ M acetate buffer solution (pH 5.0) and (**b**) 10^−1^ M acetate buffer solution (pH 5.0) containing 10^−4^ M of Cd^2+^, Pb^2+^, Cu^2+^ and Hg^2+^. Parameters applied: f (frequency) = 25 Hz; E_sw_ (applied pulse) = 20 V, E_i_–E_f_ (applied potential range) = −1.0–0.6 V. Electrodeposition parameters: E = −1.2 V for 180 s; Desorption parameters: E = 1 V for 210 s.

In Figure 5, the presence of each metal ion can be seen through a well-highlighted peak at different potentials, in accordance with literature data [63].

The electrochemical behavior of heavy metal ions has been thoroughly investigated using SWASV. Peaks are well-defined with low background noise. The recorded voltammograms demonstrate that Cd, Pb, Cu and Hg ions can be identified and quantified by this voltammetric method. The electrochemical parameters obtained with SPE/Fe_3_O_4_-CNF and SPE/Fe_3_O_4_-MWCNT by applying SWASV are presented in Table 3.

It can be seen from Table 3 that all the peaks corresponding to heavy metals in the solution have a higher intensity and are better defined when SPE/Fe_3_O_4_-CNF was used for detection compared to SPE/Fe_3_O_4_-MWCNT. This may be due to the higher affinity of Fe_3_O_4_ nanoparticles for CNFs and, therefore, a better sensitivity for heavy metals that are present in the solution. The E_pa_ values constitute crucial aspects in electrochemical analysis, signifying the preferred moments for oxidation reactions at the electrode surface. In the context of the presented research, a diversity of E_pa_ values for various metal ions on SPE/Fe_3_O_4_-CNF and SPE/Fe_3_O_4_-MWCNT electrodes has been highlighted.

The negative E_pa_ values for ions such as Cd^2+^ and Pb^2+^ indicate a propensity towards oxidation at lower potentials, while the positive values associated with Cu^2+^ and Hg^2+^ denote oxidation processes occurring at higher potentials. These variations can be attributed to structural differences in metal ions and interactions with electrode surfaces. The slight differences between the electrodes can be explained by the distinctions in the structural materials employed. It is important to emphasize that the E_pa_ values are congruent with the conclusions of previous studies, underscoring the consistency of experimental choices [63].

### 3.4. Optimization of Working Parameters

In the next step, optimization studies of the working parameters, namely, the influence of bismuth ions in the electrochemical response, the pH of the solution and the deposition time, were carried out.

**Figure 6:**
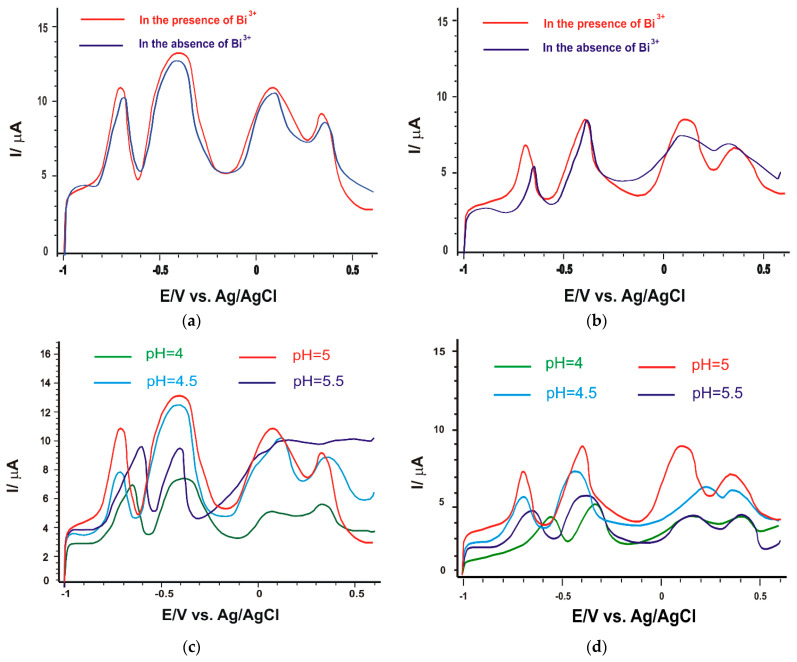

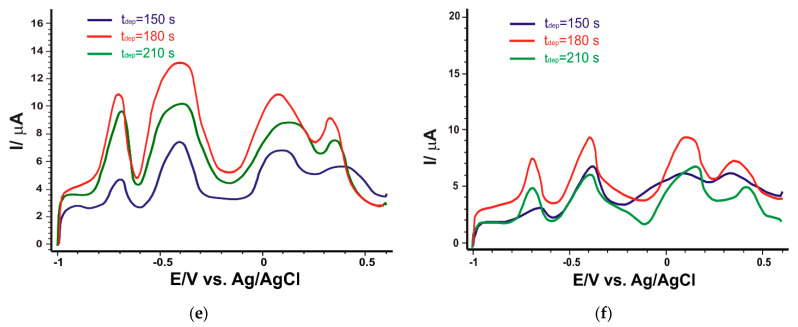
SWASV of the sensor (**a**) SPE/Fe_3_O_4_-CNF and (**b**) SPE/Fe_3_O_4_-MWCNT in 10^−4^ M heavy metal stock solution (of each metal ion) in the presence (red line) and absence of Bi (blue line); SWASV of the sensor (**c**) SPE/Fe_3_O_4_-CNF and (**d**) SPE/Fe_3_O_4_-MWCNT in the presence of Bi immersed in heavy metal stock solution at different pH values; SWASV of the sensor (**e**) SPE/Fe_3_O_4_-CNF and (**f**) SPE/Fe_3_O_4_-MWCNT in the presence of Bi immersed in heavy metal stock solution using different deposition times.

As shown in Figure 6a,b, the current intensities corresponding to each metal ion in the solution, recorded with the SPE/Fe_3_O_4_-CNF and SPE/Fe_3_O_4_-MWCNT sensors in the presence of 0.3 mg/L Bi^3+^, showed a slight increase in peak intensity for all metal ions, but also more stable signals regarding consistency and predictability over time, along with reduced background noise. When a lower concentration (0.2 mg/L) was introduced, improvements in signal fidelity were observed for only two of the metal ions (Cd^2+^ and Pb^2+^); consequently, the decision was made to use a higher concentration.

Specific interactions between the Bi^3+^ ion and the electrode surfaces can facilitate oxidation reactions associated with heavy metal ions. Additionally, signal stabilization could result from a positive influence of Bi^3+^ ions on the kinetics of electrochemical processes, thereby enhancing signal reproducibility. These are confirmed by calculating the standard deviation of the intensity values corresponding to heavy metal peaks in the presence and absence of Bi^3+^ (five recorded cyclic voltammograms) for both newly developed sensors, and the results are detailed in Table 4. This result indicates that bismuth could significantly improve the analytical performance by forming a “molten alloy” with the target metals, thus facilitating the redox process at the electrode surface [52]. Thus, both modified sensors in the presence of Bi^3+^ had improved sensitivity.

Next, the effect of pH on the current strengths in the heavy metal-containing stock solution was studied. During electrodeposition, there is a local increase in pH at the electrode surface that should not be neglected; therefore, the pH value of the buffer solution should be optimized. In this study, four pH values were studied: 4, 4.5, 5 and 5.5. According to the literature, in the application of the SWASV technique, the optimal pH range of the acetate buffer solution is 4–5.6 [76].

**Figure 7:**
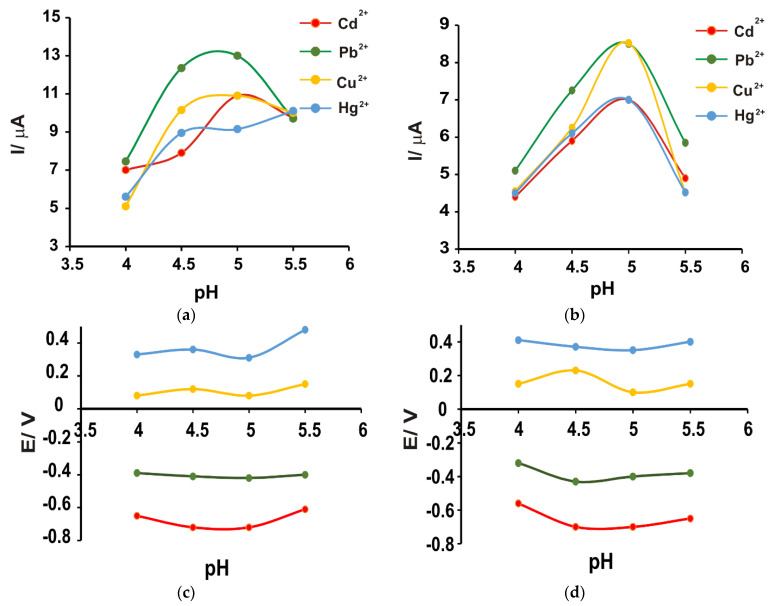
The dependence between pH and the peak currents related to the presence of metal ions in the case of (**a**) SPE/Fe_3_O_4_-CNF and (**b**) SPE/Fe_3_O_4_-MWCNT. The relationship between pH and the potential associated with the presence of a metal ion is depicted for (**c**) SPE/Fe_3_O_4_-CNF and (**d**) SPE/Fe_3_O_4_-MWCNT configurations.

As can be seen in Figure 6b,c, there is a considerable difference between the appearance of the voltammograms at the four pH values. The lower pH value resulted in currents of much lower intensity, possibly due to the protonation of the hydrophilic groups of the carbon nanofibers [78]. With increasing pH (4.5 and 5), the currents increased progressively, with a slight decrease when the pH of the solution was 5.5 (Figure 7a). The decrease in current intensity was probably due to the formation of metal complexes [78]. This behavior was observed for both sensors. In the case of the SPE/Fe_3_O_4_-CNF sensor, at pH = 5.5, although the Cd^2+^ and Pb^2+^ peaks are well-defined, the signal loses stability and the presence of Cu^2+^ and Hg^2+^ is no longer evident. Also, the potential at which heavy metal oxidation peaks occur is lower when the pH value is 5, which contributes to better sensor sensitivity. A lower potential at which oxidation peaks occur can enhance the oxidation reactions of heavy metal ions, leading to a more pronounced electrochemical signal generation, thereby contributing to improved sensor sensitivity. It is therefore considered that pH 5 is optimal for the simultaneous determination of the four heavy metal ions.

Finally, the effect of deposition time was investigated, as shown in Figure 6d,e. The current intensities increased significantly when the deposition time changed from 150 s to 180 s. However, at a longer deposition time of 210 s, the current intensity decreased obviously. This behavior may be due to a saturation effect for the bismuth film and the amount of heavy metal ions on the electrode surface. Thus, 180 s was selected as the optimal deposition time.

In Figure 7, the graphs illustrate both the relationship between pH and current intensities, as well as the dependence between pH and the potential associated with metal ions for the two modified sensors.

### 3.5. The Electrochemical Responses of the SPE/Fe_3_O_4_-CNF and SPE/Fe_3_O_4_-MWCNT Sensors in Solutions of Different Concentrations

The study continued with the realization of the calibration curves, this being an important step for the characterization of the developed voltammetric method, calculating the limits of detection (LOD) and quantification (LOQ) for each electrochemical sensor. Thus, the SWASV method was used for all electrodes modified for the detection and subsequent quantification of heavy metal ions Cd^2+^, Pb^2+^, Cu^2+^ and Hg^2+^ using the same working parameters as in the previous stage, i.e., the presence of bismuth ions, pH 5, adsorption potential and time (E = −1.2 V, time 180 s), desorption potential and time (E = 1 V, time 210 s). The concentration range studied was 0.5–80 µM. The solutions for making the calibration curve were obtained by mixing different volumes of 10^−4^ M heavy metal stock solution added in an acetate buffer solution.

**Figure 8:**
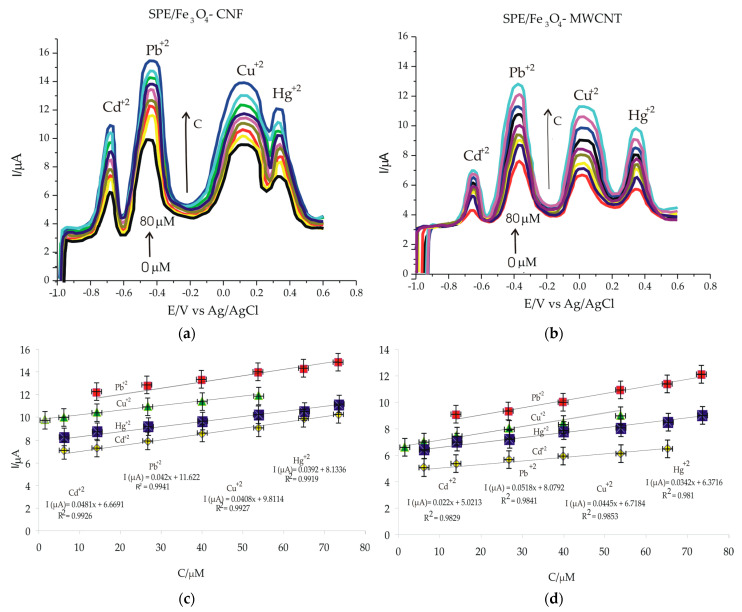
SWASV for (**a**) SPE/Fe_3_O_4_-CNF and (**b**) Fe_3_O_4_-MWCNT immersed in solutions containing the metal ions Cd^2+^, Pb^2+^, Cu^2+^ and Hg^2+^ at different concentrations: 0.5, 1.5, 5, 15, 25, 40, 55, 65, 75, 80 μM; The calibration curves and the equations of the calibration lines obtained by the electrodes (**c**) SPE/Fe_3_O_4_ -CNF and (**d**) Fe_3_O_4_-MWCNT.

Figure 8a,b shows the voltammograms obtained in standard solutions containing heavy metal ions, of different concentrations, in the 0.5–80 µM range, and Figure 8c,d shows a linear dependence between the intensity of the anodic peaks and the concentration of each metal ion in the solution to be analyzed, both for SPE/Fe_3_O_4_-CNF and for SPE/Fe_3_O_4_-MWCNT. However, superior analytical performances were obtained with the SPE/Fe_3_O_4_-CNF sensor, which shows increased anodic peak intensities compared to the SPE/Fe_3_O_4_-MWCNT sensor, due to the increased compatibility of nanoparticles for CNFs, but especially for heavy metal ions, to which the sensitivity is higher.

The linear dependence between the intensity of the peaks and their concentration in solution led to obtaining some quality linear models with coefficients between 0.98 and 1. Using the calibration equations, limits of detection (LOD) were calculated based on the relation 3 υ/m, where υ is standard deviation and m is the slope value, and limit of quantification (LOQ) was calculated according to the relation 10 υ/m. All these obtained values are included in Table 4. Comparing the two sensors performed in this study, it can be appreciated that the SPE/Fe_3_O_4_-CNF sensor is more sensitive for the detection of the studied Cd^2+^ and Hg^2+^, with lower detection and quantification limits. The superior performance of SPE/Fe_3_O_4_-CNF is due to the synergistic interaction between carbon nanofibers and Fe_3_O_4_ nanoparticles, the increased adsorption rate on the modified surface of heavy metal cations in the presence of bismuth ions [63]. Furthermore, the thin and long fibers of CNF allow for uniform dispersion in the matrix, enhancing structural stability and homogeneous distribution among Fe_3_O_4_ nanoparticles. Additionally, the long fiber of CNF improves the mechanical strength of composite materials, providing superior properties compared to shorter carbon nanotubes. These characteristics contribute to a lower detection limit and an extended linear range in the sensor’s performance.

Also, the sensors developed and characterized in this study were compared with other sensors reported in the literature for simultaneous detection of heavy metals Cd^2+^, Pb^2+^, Cu^2+^ and Hg^2+^. It is observed that the results obtained are comparable or even better than those obtained with other devices when detecting the same metal ions, as shown in Table 5.

Since the SPE/Fe_3_O_4_-CNF sensor has shown superior analytical performance, it was used for the analysis of environmental samples, namely, water samples from the Danube River.

### 3.6. Studies on the Accuracy, Reproducibility and Stability of the SPE/Fe_3_O_4_-CNF Sensor

**Figure 9:**
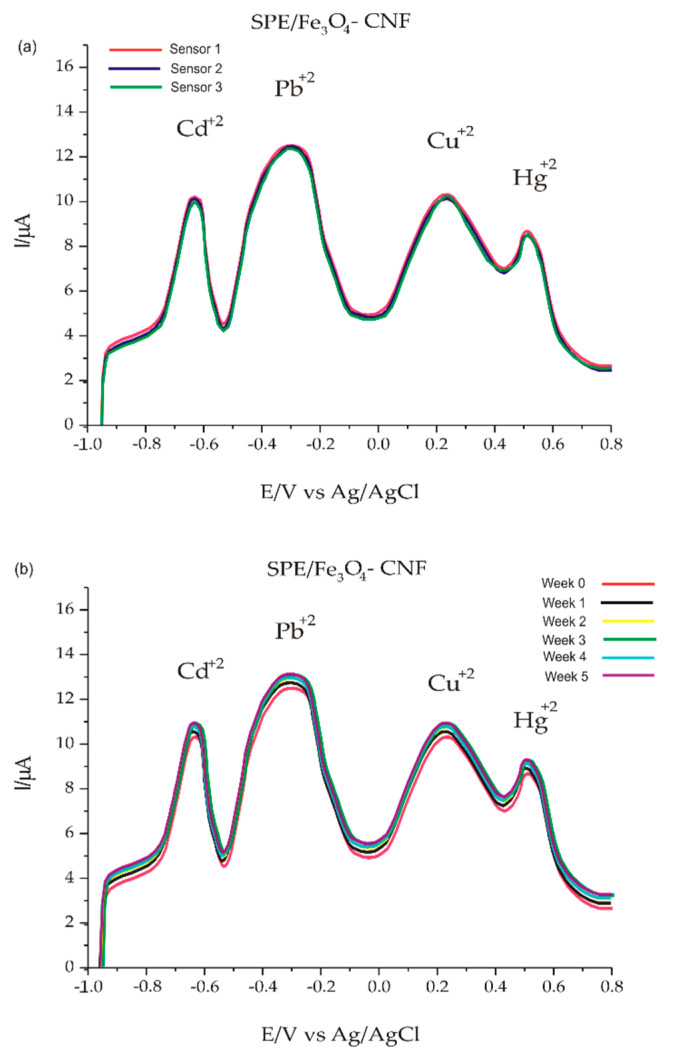
Reproducibility and stability measurements. (**a**) CV response of three SPE/Fe_3_O_4_-CNF prepared in similar conditions for the detection of 5 × 10^−6^ M metal ions Cd^2+^, Pb^2+^, Cu^2+^ and Hg^2+^ in 10^−1^ M acetate buffer solution at pH 5; (**b**) the CV response of SPE/Fe_3_O_4_-CNF for the detection of 5 × 10^−6^ M metal ions Cd^2+^, Pb^2+^, Cu^2+^ and Hg^2+^ in 10^−1^ M acetate buffer solution at pH 5 for five weeks.

Studies on the accuracy and precision of the voltametric method were carried out in a solution of Cd^2+^, Pb^2+^, Cu^2+^ and Hg^2+^ containing the four cations in equal concentrations of 5 × 10^−6^ M in 10^−1^ M acetate buffer solution of pH 5. Evaluation of the accuracy parameters was performed over a period of 10 consecutive days (inter-day) and also over a period of 1 day at different times at an interval of 3 h (intra-day). The relative standard deviation (RSD) obtained was reported in Table 6. Both short-term and long-term, good stability of Fe_3_O_4_-CNF electrode was found by the SWASV method, as the initial signal was preserved at 98.3% (Cd^2+^), 95.24% (Pb^2+^), 94.12% (Cu^2+^) and 93.85% (Hg^2+^), illustrated in Figure 9. The coefficients of variation are not lower than 5% and the results did not demonstrate significant differences between the anodic currents recorded on different days; this demonstrates the fact that the sensor is stable and can be used in electroanalysis.

The reproducibility of Fe_3_O_4_-CNF-modified sensor fabrication was studied in 5 × 10^−6^ M Cd^2+^, Pb^2+^, Cu^2+^ and Hg^2+^ concentration solutions. Three identically constructed sensors on the same day were immersed in the solution to be analyzed. Therefore, the sensor fabrication process is suitable for obtaining sensors with analytical performance adequate for practical applications.

### 3.7. Interference Studies

Since some metal ions have a close reduction potential, it was considered in this study step to evaluate the selectivity of the SPE/Fe_3_O_4_-CNF sensor towards metal ions in complex samples, where, in addition to the four metal ions studied, other metal ions such as Co^2+^, Ni^2+^, Mn^2+^ and Zn^2+^ were added.

**Figure 10:**
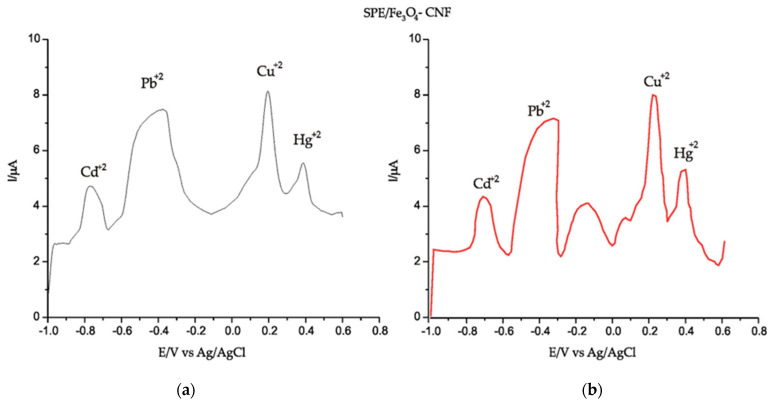
SWASV for SPE/Fe_3_O_4_-CNF immersed in solutions containing the metal ions Cd^2+^, Pb^2+^, Cu^2+^ and Hg^2+^ (**a**) and in solution with interfering ions Co^2+^, Ni^2+^, Mn^2+^ and Zn^2+^ (**b**) at concentration 2 × 10^−4^ M in 10^−1^ M acetate buffer solution (pH 5.0).

There were insignificant changes in the peak currents of the studied ions in the presence of interfering ions. In this sense Figure 10 shows the voltammograms recorded in the presence of interfering ions Co^2+^, Ni^2+^, Mn^2+^ and Zn^2+^. The results obtained indicate variations in concentrations of less than 2% for all ions studied (Table 7).

Analyzing these experimental results, it can be concluded that the SWASV method is suitable for the analysis of metal ions Cd^2+^, Pb^2+^, Cu^2+^ and Hg^2+^ in the presence of others, such as Co^2+^, Ni^2+^, Mn^2+^ and Zn^2+^.

### 3.8. Determination of Metal Ions in Water Samples

The applicability of the SPE/Fe_3_O_4_-CNF sensor was demonstrated by analyzing water samples taken from the Danube River from five different locations (Isaccea, Galati, Prut, Reni and Tulcea) on the same day.

The measurements with the developed sensor were performed by the SWASV method, using the same working parameters (f = 25 Hz, E_sw_ = 20 V, E_i_–E_f_ = −1.0–0.6 V, absorption potential E = −1.2 V in 180 s and a desorption potential of 1 V in a time of 210 s) in order to quantify metal ions (Cd^2+^, Pb^2+^, Cu^2+^ and Hg^2+^), and, for each sample, we analyzed four replicates.

**Figure 11:**
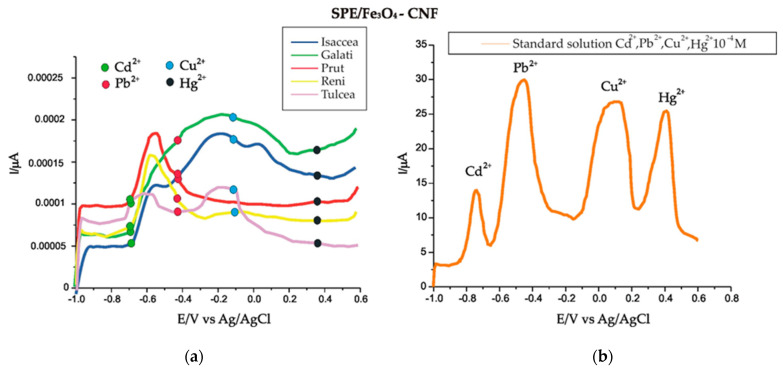
(**a**) SWASV of the SPE/Fe_3_O_4_-CNF sensor immersed in water samples taken on the same day from different points of the Danube River. (**b**) SWASV of the SPE/Fe_3_O_4_-CNF sensor immersed in standard solution 10^−4^ M Cd^2+^, Pb^2+^, Cu^2+^ and Hg^2+^ in 10^−1^ M acetate buffer solution (pH 5.0).

Figure 11a illustrates the voltammograms recorded with the SPE/Fe_3_O_4_-CNF sensor for the five water samples taken from different points.

Using the measured anodic peak intensities at the specific potential of each metal ion and the equation of the calibration line, each concentration was calculated, as shown in Table 8.

According to the intensity of the anodic peaks and the concentrations obtained for each metal ion (Table 8), the values fall within the maximum concentration limits laid out in currently enforced legislation. It can be emphasized that the SPE/Fe_3_O_4_-CNF sensor is sensitive to the detection of heavy metal ions included in this research and can be successfully used in surface water analysis.

### 3.9. Validation of Results by the Standard Addition Method

The sensor performance was validated using the standard addition method. Thus, in the already analyzed real samples (Isaccea), well-known concentrations of metal ions were added using 10^−6^ M standard solutions. The final concentrations of metal ions in the real samples were calculated, and the recoveries ranged between 96.89 and 103.41%, while the RSD (relative standard deviation) did not exceed 5%. These results indicate an optimal precision of the newly constructed sensor for detecting heavy metal ions in water samples. The results are presented in Table 9.

The advantages of the prepared sensors are low cost, short preparation and result acquisition time, accuracy and portability for in situ sample measurement with minimal water sample treatment.

## 4. Conclusions

Fe_3_O_4_ nanoparticles with carbon nanofibers and multilayer carbon nanotubes were successfully synthesized and deposited on screen-printed carbon electrodes by the CA method. SPE/Fe_3_O_4_-CNF and SPE/Fe_3_O_4_-MWCNT sensors were prepared for the detection of heavy metal ions Cd^2+^, Pb^2+^, Cu^2+^ and Hg^2+^ both in standard solutions and in surface water samples. The best analytical performances were obtained with the SPE/Fe_3_O_4_-CNF sensor, compared to the SPE/Fe_3_O_4_-MWCNT sensor, by the SWASV method, a method with a series of advantages such as precision, reproducibility, stability, low cost and simplicity. Due to the fast response, low detection limit and good sensitivity, this sensor proves to be useful for the analysis of complex samples containing different heavy metal ions. All these positive aspects make this new sensor a promising monitoring tool for determining the quality of water or other environmental samples.

## Figures and Tables

**Table 1:** Main electrochemical parameters obtained from voltammograms of sensors immersed in an aqueous solution with K_4_[Fe(CN)_6_] 10^−3^ M and KCl 10^−1^ M.

Electrode	E_pa_ ^1^ (V)	E_pc_ ^2^ (V)	E_1/2_ ^3^ (V)	I_pa_ ^4^ (µA)	I_pc_ ^5^ (µA)	ΔE ^6^	I_pc_/I_pa_
SPE	0.201	0.045	0.123	19.85	−24.35	0.156	1.22
SPE/CNF	0.195	0.015	0.105	25.67	−30.85	0.180	1.20
SPE/MWCNT	0.300	0.135	0.217	61.75	−71.08	0.165	1.15
SPE/Fe_3_O_4_-CNF	0.274	0.126	0.200	94.11	−83.89	0.148	0.89
SPE/Fe_3_O_4_-MWCNT	0.265	−0.126	0.195	62.11	−74.78	0.391	1.20

^1^ potential of the anodic peak; ^2^ potential of the cathodic peak; ^3^ half-wave potential; ^4^ current of the anodic peak; ^5^ current of the cathodic peak; ^6^ ΔE = E_pa_ − E_pc_.

**Table 2:** I_pa_ vs. v^1/2^ and log I_pa_ vs. log v linear regression equations, R^2^, geometrical areas, active surface areas and the roughness factors of all the modified SPEs studied.

Electrode	Linear Equation I_pa_ vs. v^1/2^	R^2^	Linear Equation log I_pa_ vs. log v	R^2^	Points	Geometric Area (cm^2^)	Active Area (cm^2^)	Roughness Factor
SPE	I (A) = 6.64 × 10^−5^ v^1/2^ (V·s^−1^)^1/2^ + 4.61 × 10^−6^	0.9861	log I_pa_ = 0.4614 log v − 4.1402	0.9865	5	0.125	0.032	0.25
SPE/CNF	I (A) = 7.79 × 10^−5^ v^1/2^ (V·s^−1^)^1/2^ + 1.85 × 10^−6^	0.9972	log I_pa_ = 0.4891log v − 4.0953	0.9975			0.039	0.31
SPE/MWCNT	I (A) = 1.77 × 10^−4^ v^1/2^ (V·s^−1^)^1/2^ + 8.83 × 10^−6^	0.9893	log I_pa_ = 0.4688log v − 3.7283	0.9905			0.086	0.69
SPE/Fe_3_O_4_-CNF	I (A) = 5.43 × 10^−4^ v^1/2^ (V·s^−1^)^1/2^ + 7.78 × 10^−5^	0.9999	log I_pa_ = 0.7341log v − 3.282	0.9972			0.265	2.12
SPE/Fe_3_O_4_-MWCNT	I (A) = 3.72 × 10^−4^ v^1/2^ (V·s^−1^)^1/2^ + 5.35 × 10^−5^	0.9979	log I_pa_ = 0.7524log v − 3.4367	0.9914			0.181	1.44

**Table 3:** The values of the parameters obtained from square-wave voltamograms of SPE/Fe_3_O_4_-CNF and SPE/Fe_3_O_4_-MWCNT in 10^−4^ M metallic ions (the electrolyte support was 10^−1^ M acetate buffer at pH 5.0).

Electrode	Metallic Ion	E_pa_ (V)	I_pa_ (µA)
**SPE/Fe_3_O_4_-CNF**	Cd^2+^	−0.696	10.150
	Pb^2+^	−0.423	12.681
	Cu^2+^	0.106	10.607
	Hg^2+^	0.362	8.581
**SPE/Fe_3_O_4_-MWCNT**	Cd^2+^	−0.648	5.545
	Pb^2+^	−0.374	8.783
	Cu^2+^	0.092	7.670
	Hg^2+^	0.341	7.213

**Table 4:** Standard deviations of peak intensities for heavy metals in the presence and absence of Bi^3+^ for SPE/Fe_3_O_4_-CNF and SPE/Fe_3_O_4_-MWCNT.

σ (Standard Deviation)								
Sensor	Cd^2+^		Pb^2+^		Cu^2+^		Hg^2+^	
	In Presence of Bi^3+^	In Absence of Bi^3+^	In Presence of Bi^3+^	In Absence of Bi^3+^	In Presence of Bi^3+^	In Absence of Bi^3+^	In Absence of Bi^3+^	In Absence of Bi^3+^
SPE/Fe_3_O_4_-CNF	0.00376	0.00646	0.00415	0.01870	0.00178	0.00719	0.00788	0.01036
SPE/Fe_3_O_4_-MWCNT	0.00618	0.01	0.00291	0.01045	0.00364	0.00828	0.00985	0.01848

**Table 5:** Comparative results of the performance of the electrodes reported in this study with those in the literature.

Electrode Material	Metal Ion	Method	Linear Range (μM)	LOD (μM)	LOQ (μM)	Reference
SPE/Fe_3_O_4_-CNF	Cd^2+^	SWASV	6–80	0.0615	0.2065	In this study
	Pb^2+^		14–80	0.0154	0.0514	
	Cu^2+^		1–60	0.0320	0.1066	
	Hg^2+^		6–80	0.0148	0.0493	
SPE/Fe_3_O_4_-MWCNT	Cd^2+^	SWASV	6–70	0.2719	0.9064	In this study
	Pb^2+^		14–80	0.3187	1.0623	
	Cu^2+^		1–60	1.0436	3.4789	
	Hg^2+^		6–80	0.9076	3.0256	
SnO_2_ modified electrode	Pb^2+^	SWASV	0.3–0.1	0.0104	0.0259	[79]
Fe_3_O_4_-chitosan modified GCE (glassy carbon electrode)	Cd^2+^	SWASV	1.2–1.7	0.0392	0.1306	[80]
	Pb^2+^		0.1–1.4	0.0422	0.1406	
	Cu^2+^		0.3–1.2	0.0967	0.3223	
	Hg^2+^		0.4–1.2	0.0957	0.3191	
GCE/CN-polymer	Cd^2+^	DPASV	0.04–0.27	2.27	7.56	[81]
	Pb^2+^		0.002–0.15	0.8	2.66	
Fe_3_O_4_@SiO_2_	Cd^2+^	DPASV	0.1–100	0.0561	0.1871	[82]
	Pb^2+^		0.1–80	0.0165	0.0550	
	Cu^2+^		0.1–80	0.0794	0.2642	
	Hg^2+^		0.1–100	0.0567	0.1891	

SWASV—square-wave anodic stripping voltammetry, DPASV—differential pulse anodic stripping voltammetry.

**Table 6:** The recovery test of the electrode for accuracy and reproducibility of the method.

Metallic Ion		Cd^2+^	Pb^2+^	Cu^2+^	Hg^2+^
Accuracy (RSD%)	Intra-day	2.05	2.19	2.87	2.42
	Inter-day	3.15	3.43	3.98	3.44
Reproducibility (RSD%)		2.10	2.07	2.66	2.37

**Table 7:** The results obtained for the quantitative determination of metal ions in the presence of interfering ions.

Sample	[Cd^2+^,Pb^2+^, Cu^2+^, Hg^2+^]/M	[Co^2+^,Ni^2+^, Mn^2+^, Zn^2+^]/M	[Cd^2+^,Pb^2+^, Cu^2+^, Hg^2+^]/M Found	Recovery (RSD) (%)
1	2 × 10^−4^	2 × 10^−4^	2.01 × 10^−4^	100.50 ± 0.35
	3 × 10^−4^	2 × 10^−4^	3.02 × 10^−4^	100.66 ± 0.47
	4 × 10^−4^	2 × 10^−4^	4.01 × 10^−4^	100.25 ± 0.17
	5 × 10^−4^	2 × 10^−4^	5.01 × 10^−4^	100.20 ± 0.14
2	2 × 10^−4^	3 × 10^−4^	2.04 × 10^−4^	102.00 ± 1.4
	3 × 10^−4^	3 × 10^−4^	2.98 × 10^−4^	99.33 ± 0.47
	4 × 10^−4^	3 × 10^−4^	3.99 × 10^−4^	99.75 ± 0.17
	5 × 10^−4^	3 × 10^−4^	5.03 × 10^−4^	100.60 ± 0.42
3	2 × 10^−4^	4 × 10^−4^	2.02 × 10^−4^	101.00 ± 0.70
	3 × 10^−4^	4 × 10^−4^	3.04 × 10^−4^	101.33 ± 0.93
	4 × 10^−4^	4 × 10^−4^	3.89 × 10^−4^	97.25 ± 1.97
	5 × 10^−4^	4 × 10^−4^	4.95 × 10^−4^	99.00 ± 0.71
4	2 × 10^−4^	5 × 10^−4^	1.97 × 10^−4^	98.50 ± 1.06
	3 × 10^−4^	5 × 10^−4^	3.06 × 10^−4^	102.00 ± 1.40
	4 × 10^−4^	5 × 10^−4^	3.95 × 10^−4^	98.75 ± 0.88
	5 × 10^−4^	5 × 10^−4^	5.01 × 10^−4^	100.20 ± 0.14
5	2 × 10^−4^	6 × 10^−4^	2.03 × 10^−4^	101.50 ± 1.05
	3 × 10^−4^	6 × 10^−4^	2.99 × 10^−4^	99.66 ± 0.23
	4 × 10^−4^	6 × 10^−4^	3.94 × 10^−4^	98.50 ± 1.06
	5 × 10^−4^	6 × 10^−4^	4.95 × 10^−4^	99.00 ± 0.71

**Table 8:** Heavy metal ion concentration values obtained with SPE/Fe_3_O_4_-CNF immersed in water samples taken on the same day from different points on the Danube River.

Metals	C (μg/L)					USEPA [83] (μg/L)	WHO [83] (μg/L)
	Isaccea	Galati	Prut	Reni	Tulcea		
Cd^2+^	0.1171	0.1682	0.2360	0.1612	0.2827	5	3
Pb^2+^	0.6314	0.8633	0.8880	0.5525	0.4045	15	10
Cu^2+^	0.2337	0.2803	0.1541	0.1248	0.0967	1300	2000
Hg^2+^	0.7061	0.8289	0.5219	0.3689	0.2568	2	1

**Table 9:** Quantitative results obtained for the detection of metallic ions by the standard addition method.

Sample	Cd^2+^ (μg/L) Initial	Cd^2+^ (μg/L) Added	Cd^2+^ (μg/L) Found	Recovery (%)
Isaccea	0.1171	0.1	0.2221	102.30 ± 1.60
		0.3	0.4061	97.36 ± 1.88
		0.5	0.6211	100.64 ± 0.45
	**Pb^2+^ (μg/L)** **Initial**	**Pb^2+^ (μg/L)** **Added**	**Pb^2+^ (μg/L)** **Found**	**Recovery (%)**
	0.6314	0.1	0.7421	101.46 ± 1.02
		0.3	0.9025	96.89 ± 2.22
		0.5	1.122	99.17 ± 0.58
	**Cu^2+^ (μg/L)** **Initial**	**Cu^2+^ (μg/L)** **Added**	**Cu^2+^ (μg/L)** **Found**	**Recovery (%)**
	0.2337	0.1	0.3451	103.41 ± 2.37
		0.3	0.5301	99.32 ± 0.47
		0.5	0.7425	101.19 ± 084
	**Hg^2+^ (μg/L)** **Initial**	**Hg^2+^ (μg/L)** **Added**	**Hg^2+^ (μg/L)** **Found**	**Recovery (%)**
	0.7061	0.1	0.825	102.34 ± 1.63
		0.3	1.026	101.97 ± 1.38
		0.5	1.202	99.66 ± 0.24

## Data Availability

Data are contained within the article.
